# Noether's symmetry and conserved quantity for a time-delayed Hamiltonian system of Herglotz type

**DOI:** 10.1098/rsos.180208

**Published:** 2018-10-03

**Authors:** Yi Zhang

**Affiliations:** College of Civil Engineering, Suzhou University of Science and Technology, Suzhou, Jiangsu 215011, People's Republic of China

**Keywords:** time-delayed Hamiltonian system, variational problem of Herglotz type, Noether symmetry, conserved quantity

## Abstract

The variational problem of Herglotz type and Noether's theorem for a time-delayed Hamiltonian system are studied. Firstly, the variational problem of Herglotz type with time delay in phase space is proposed, and the Hamilton canonical equations with time delay based on the Herglotz variational problem are derived. Secondly, by using the relationship between the non-isochronal variation and the isochronal variation, two basic formulae of variation of the Hamilton–Herglotz action with time delay in phase space are derived. Thirdly, the definition and criterion of the Noether symmetry for the time-delayed Hamiltonian system are established and the corresponding Noether's theorem is presented and proved. The theorem we obtained contains Noether's theorem of a time-delayed Hamiltonian system based on the classical variational problem and Noether's theorem of a Hamiltonian system based on the variational problem of Herglotz type as its special cases. At the end of the paper, an example is given to illustrate the application of the results.

## 1. Introduction

It is well known that variational principles play an important role in the fields of mechanics, physics and engineering, etc. However, with the classical variational principle it is difficult to describe non-conservative or dissipative physical processes. Herglotz proposed a class of generalized variational principle, which generalizes the classical variational principle by defining its functional whose extreme is sought by a differential equation [1]. Compared with the classical variational principle, the generalized variational principle of Herglotz type can give a variational description of a non-conservative dynamic system. It can describe not only all dynamic processes that the classical variational principle can, but also many others for which the classical variational principle is not applicable [2]. The principle is a promotion of classical variational principle. In 2002, Georgieva and Guenther proposed and proved the first Noether-type theorem for the generalized variational principle of Herglotz [2], and extended the results to one with several independent variables [3]. Donchev applied the generalized variational principle of Herglotz type and its Noether's theorem to Böcher equation and nonlinear Schrödinger equation, etc. that these equations do not have a variational description with the classical variational principle [4]. Santos *et al*. studied the variational problem of Herglotz type with time delay and obtained the corresponding Noether's theorem [5]. The research on the variation problems of Herglotz type and the corresponding symmetries and the conserved quantities has attracted strong attention of scholars and obtained a series of important results [6–14]. In this paper, we will extend the variational problem of Herglotz type to a time-delayed Hamiltonian system and study the Noether symmetry and the conserved quantity for the system based on the generalized variational principle of Herglotz type.

## 2. Generalized variational principle for a time-delayed Hamiltonian system of Herglotz type

According to the generalized variational principle of Herglotz [1,2], the variational problem of Herglotz type with time delay can be defined as follows:

Determine the trajectories *q*_*s*_(*t*), *p*_*s*_(*t*) to extremize the value *z*(*t*_1_) where the functional *z* is defined by the differential equation

2.1 $$\dot{z}(t)=p_{s}(t){\dot{q}}_{s}(t)+p_{s}(t-\tau ){\dot{q}}_{s}(t-\tau )-H(t,q_{s}(t),p_{s}(t),q_{s}(t-\tau ),p_{s}(t-\tau ),z(t))$$

and satisfying a given initial condition

2.2 $$z(t){|}_{t=t_{0}}=z_{0}$$

and boundary conditions

2.3 $$q_{s}(t)=f_{s}(t),p_{s}(t)=g_{s}(t),t\in [t_{0}-\tau ,t_{0}],(s=1,2,\cdots ,n)$$

2.4 $$\;\mathrm{and}\;q_{s}(t)=q_{s}(t_{1}),p_{s}(t)=p_{s}(t_{1}),t=t_{1},(s=1,2,\cdots ,n),$$

where

$$H(t,q_{s}(t),p_{s}(t),q_{s}(t-\tau ),p_{s}(t-\tau ),z(t))≜H(t,q_{s},p_{s},q_{s\tau },p_{s\tau },z)$$

can be called the Hamiltonian for the variational problem of Herglotz type with time delay; *τ* is a given positive real number such that $\tau <t_{1}-t_{0}$; $f_{s}(t)$, $g_{s}(t)$ are given piecewise smooth functions on the interval $\left[t_{0}-\tau ,t_{0}\right]$; $q_{s}(t_{1}),p_{s}(t_{1}),z_{0}$ are fixed real numbers. Here and later, we use the Einstein summation convention on repeated indices.

The functional *z* determined by formula (2.1) can be called the Hamilton–Herglotz action with time delay. The above variational problem can be referred to as the generalized variational principle for a time-delayed Hamiltonian system of Herglotz type.

## 3. Hamilton canonical equations with time delay based on the variational problem of Herglotz type

The isochronal variation of the differential equation (2.1) gives

3.1 $$\delta \dot{z}={\dot{q}}_{s}\delta p_{s}+p_{s}\delta {\dot{q}}_{s}+{\dot{q}}_{s\tau }\delta p_{s\tau }+p_{s\tau }\delta {\dot{q}}_{s\tau }-\frac{\partial H}{\partial q_{s}}\delta q_{s}-\frac{\partial H}{\partial p_{s}}\delta p_{s}-\frac{\partial H}{\partial q_{s\tau }}\delta q_{s\tau }-\frac{\partial H}{\partial p_{s\tau }}\delta p_{s\tau }-\frac{\partial H}{\partial z}\delta z.$$

Using the commutative relation $\delta \dot{z}=(d/dt)\delta z$, we can write equation (3.1) as

3.2 $$\frac{d}{dt}\delta z=A-\frac{\partial H}{\partial z}\delta z,$$

where

3.3 $$A={\dot{q}}_{s}\delta p_{s}+p_{s}\delta {\dot{q}}_{s}+{\dot{q}}_{s\tau }\delta p_{s\tau }+p_{s\tau }\delta {\dot{q}}_{s\tau }-\frac{\partial H}{\partial q_{s}}\delta q_{s}-\frac{\partial H}{\partial p_{s}}\delta p_{s}-\frac{\partial H}{\partial q_{s\tau }}\delta q_{s\tau }-\frac{\partial H}{\partial p_{s\tau }}\delta p_{s\tau }.$$

The solution of equation (3.2) is

3.4 $$\delta z(t)\exp\left(\int_{t_{0}}^{t}\frac{\partial H}{\partial z}d\theta \right)-\delta z(t_{0})=\int_{t_{0}}^{t}A\exp\left(\int_{t_{0}}^{t}\frac{\partial H}{\partial z}d\theta \right)dt.$$

From the initial condition (2.2), and noting that z(t) reaches its extreme value at $t=t_{1}$, we have

3.5 $$\delta z(t_{1})=\delta z(t_{0})=0.$$

As the equation (3.4) holds for all $t\in [t_{0},t_{1}]$, particularly, take $t=t_{1}$, we obtain

3.6 $$\begin{array}{rl} & \int_{t_{0}}^{t_{1}}\exp\left(\int_{t_{0}}^{t}\frac{\partial H}{\partial z}d\theta \right)[{\dot{q}}_{s}\delta p_{s}+p_{s}\delta {\dot{q}}_{s}+{\dot{q}}_{s\tau }\delta p_{s\tau }+p_{s\tau }\delta {\dot{q}}_{s\tau }\\ & -\frac{\partial H}{\partial q_{s}}\delta q_{s}-\frac{\partial H}{\partial p_{s}}\delta p_{s}-\frac{\partial H}{\partial q_{s\tau }}\delta q_{s\tau }-\frac{\partial H}{\partial p_{s\tau }}\delta p_{s\tau }]dt. \end{array}$$

By performing a linear change of variable for the terms involving time delay in equation (3.6), and considering the boundary conditions (2.3), we have

3.7 $$\begin{array}{rl} & \int_{t_{0}}^{t_{1}}\lambda (t)\left[{\dot{q}}_{s\tau }(t)\delta p_{s\tau }(t)+p_{s\tau }(t)\delta {\dot{q}}_{s\tau }(t)-\frac{\partial H}{\partial q_{s\tau }}(t)\delta q_{s\tau }(t)-\frac{\partial H}{\partial p_{s\tau }}(t)\delta p_{s\tau }(t)\right]dt\\ & \;=\int_{t_{0}-\tau }^{t_{1}-\tau }\lambda (\varphi +\tau )[{\dot{q}}_{s\tau }(\varphi +\tau )\delta p_{s\tau }(\varphi +\tau )+p_{s\tau }(\varphi +\tau )\delta {\dot{q}}_{s\tau }(\varphi +\tau )\\ & \;-\frac{\partial H}{\partial q_{s\tau }}(\varphi +\tau )\delta q_{s\tau }(\varphi +\tau )-\frac{\partial H}{\partial p_{s\tau }}(\varphi +\tau )\delta p_{s\tau }(\varphi +\tau )]d\varphi \\ & \;=\int_{t_{0}}^{t_{1}-\tau }\lambda (\varphi +\tau )[{\dot{q}}_{s\tau }(\varphi +\tau )\delta p_{s}(\varphi )+p_{s\tau }(\varphi +\tau )\delta {\dot{q}}_{s}(\varphi )\\ & \;-\frac{\partial H}{\partial q_{s\tau }}(\varphi +\tau )\delta q_{s}(\varphi )-\frac{\partial H}{\partial p_{s\tau }}(\varphi +\tau )\delta p_{s}(\varphi )]d\varphi , \end{array}\}$$

where $\lambda (t)=\exp\left(\int_{t_{0}}^{t}\left(\partial H/\partial z\right)d\theta \right)$. Substituting equation (3.7) into equation (3.6), we obtain

3.8 $$\begin{array}{rl} & \int_{t_{0}}^{t_{1}-\tau }\{\lambda (t)\left[{\dot{q}}_{s}(t)\delta p_{s}(t)+p_{s}(t)\delta {\dot{q}}_{s}(t)-\frac{\partial H}{\partial q_{s}}(t)\delta q_{s}(t)-\frac{\partial H}{\partial p_{s}}(t)\delta p_{s}(t)\right]\\ & +\lambda (t+\tau )\left[{\dot{q}}_{s\tau }(t+\tau )\delta p_{s}(t)+p_{s\tau }(t+\tau )\delta {\dot{q}}_{s}(t)-\frac{\partial H}{\partial q_{s\tau }}(t+\tau )\delta q_{s}(t)-\frac{\partial H}{\partial p_{s\tau }}(t+\tau )\delta p_{s}(t)\right]\}dt\\ & +\int_{t_{1}-\tau }^{t_{1}}\lambda (t)\left[{\dot{q}}_{s}(t)\delta p_{s}(t)+p_{s}(t)\delta {\dot{q}}_{s}(t)-\frac{\partial H}{\partial q_{s}}(t)\delta q_{s}(t)-\frac{\partial H}{\partial p_{s}}(t)\delta p_{s}(t)\right]dt=0. \end{array}\}$$

Making integration by parts for the terms corresponding to $\delta q_{s}$ and $\delta p_{s}$ in equation (3.12), and using the conditions (2.3) and (2.4), we have

3.9 $$\begin{array}{rl} & \int_{t_{0}}^{t_{1}-\tau }\{\left[-\lambda (t)\frac{\partial H}{\partial q_{s}}(t)-\lambda (t+\tau )\frac{\partial H}{\partial q_{s\tau }}(t+\tau )\right]\delta q_{s}(t)\\ & +[\lambda (t)\left({\dot{q}}_{s}(t)-\frac{\partial H}{\partial p_{s}}(t)\right)+\lambda (t+\tau )\left({\dot{q}}_{s\tau }(t+\tau )-\frac{\partial H}{\partial p_{s\tau }}(t+\tau )\right)]\delta p_{s}(t)\}dt\\ & {=-\{\delta q_{s}(t)\int_{t}^{t_{1}-\tau }[-\lambda (\theta )\frac{\partial H}{\partial q_{s}}(\theta )-\lambda (\theta +\tau )\frac{\partial H}{\partial q_{s\tau }}(\theta +\tau )]d\theta \}|}_{t_{0}}^{t_{1}-\tau }\\ & +\int_{t_{0}}^{t_{1}-\tau }\delta {\dot{q}}_{s}(t)\{\int_{t_{1}}^{t_{1}-\tau }\left[-\lambda (\theta )\frac{\partial H}{\partial q_{s}}(\theta )-\lambda (\theta +\tau )\frac{\partial H}{\partial q_{s\tau }}(\theta +\tau )\right]d\theta \}dt\\ & -\{\delta p_{s}(t)\int_{t}^{t_{1}-\tau }[\lambda (\theta )\left({\dot{q}}_{s}(\theta )-\frac{\partial H}{\partial p_{s}}(\theta )\right)+{\lambda (\theta +\tau )\left({\dot{q}}_{s\tau }(\theta +\tau )-\frac{\partial H}{\partial p_{s\tau }}(\theta +\tau )\right)]d\theta \}|}_{t_{0}}^{t_{1}-\tau }\\ & +\int_{t_{0}}^{t_{1}-\tau }\delta {\dot{\;p}}_{s}(t)\{\int_{t}^{t_{1}-\tau }[\lambda (\theta )\left({\dot{q}}_{s}(\theta )-\frac{\partial H}{\partial p_{s}}(\theta )\right)+\lambda (\theta +\tau )\left({\dot{q}}_{s\tau }(\theta +\tau )-\frac{\partial H}{\partial p_{s\tau }}(\theta +\tau )\right)]d\theta \}dt\\ & =\int_{t_{0}}^{t_{1}-\tau }\delta {\dot{q}}_{s}(t)\{\int_{t}^{t_{1}-\tau }\left[-\lambda (\theta )\frac{\partial H}{\partial q_{s}}(\theta )-\lambda (\theta +\tau )\frac{\partial H}{\partial q_{s\tau }}(\theta +\tau )\right]d\theta \}dt\\ & +\int_{t_{0}}^{t_{1}-\tau }\delta {\dot{\;p}}_{s}(t)\{\int_{t}^{t_{1}-\tau }[\lambda (\theta )\left({\dot{q}}_{s}(\theta )-\frac{\partial H}{\partial p_{s}}(\theta )\right)+\lambda (\theta +\tau )\left({\dot{q}}_{s\tau }(\theta +\tau )-\frac{\partial H}{\partial p_{s\tau }}(\theta +\tau )\right)]d\theta \}dt \end{array}\}$$

and

3.10 $$\begin{array}{rl} & \int_{t_{1}-\tau }^{t_{1}}\left[-\lambda (t)\frac{\partial H}{\partial q_{s}}(t)\delta q_{s}(t)+\lambda (t)\left({\dot{q}}_{s}(t)-\frac{\partial H}{\partial p_{s}}(t)\right)\delta p_{s}(t)\right]dt\\ & \;{=\left\{\delta q_{s}(t)\int_{t_{1}-\tau }^{t}\left[-\lambda (\theta )\frac{\partial H}{\partial q_{s}}(\theta )\right]d\theta \right\}|}_{t_{1}-\tau }^{t_{1}}+{\left\{\delta p_{s}(t)\int_{t_{1}-\tau }^{t}\left[\lambda (\theta )\left({\dot{q}}_{s}(\theta )-\frac{\partial H}{\partial p_{s}}(\theta )\right)\right]d\theta \right\}|}_{t_{1}-\tau }^{t_{1}}\\ & \;-\int_{t_{1}-\tau }^{t_{1}}\delta {\dot{q}}_{s}(t)\left\{\int_{t_{1}-\tau }^{t}\left[-\lambda (\theta )\frac{\partial H}{\partial q_{s}}(\theta )\right]d\theta \right\}dt\\ & \;-\int_{t_{1}-\tau }^{t_{1}}\delta {\dot{\;p}}_{s}(t)\left\{\int_{t_{1}-\tau }^{t}\left[\lambda (\theta )\left({\dot{q}}_{s}(\theta )-\frac{\partial H}{\partial p_{s}}(\theta )\right)\right]d\theta \right\}dt\\ & \;=-\int_{t_{1}-\tau }^{t_{1}}\delta {\dot{q}}_{s}(t)\left\{\int_{t_{1}-\tau }^{t}\left[-\lambda (\theta )\frac{\partial H}{\partial q_{s}}(\theta )\right]d\theta \right\}dt\\ & \;-\int_{t_{1}-\tau }^{t_{1}}\delta {\dot{\;p}}_{s}(t)\left\{\int_{t_{1}-\tau }^{t}\left[\lambda (\theta )\left({\dot{q}}_{s}(\theta )-\frac{\partial H}{\partial p_{s}}(\theta )\right)\right]d\theta \right\}dt. \end{array}\}$$

Substituting formulae (3.9) and (3.10) into equation (3.8), we obtain

3.11 $$\begin{array}{rl} & \int_{t_{0}}^{t_{1}-\tau }\delta {\dot{q}}_{s}(t)\{\lambda (t)p_{s}(t)+\lambda (t+\tau )p_{s\tau }(t+\tau )+\int_{t}^{t_{1}-\tau }[-\lambda (\theta )\frac{\partial H}{\partial q_{s}}(\theta )\\ & \;-\lambda (\theta +\tau )\frac{\partial H}{\partial q_{s\tau }}(\theta +\tau )]d\theta \}dt+\int_{t_{0}}^{t_{1}-\tau }\delta {\dot{\;p}}_{s}(t)\{\int_{t}^{t_{1}-\tau }[\lambda (\theta )\left({\dot{q}}_{s}(\theta )-\frac{\partial H}{\partial p_{s}}(\theta )\right)\\ & \;+\lambda (\theta +\tau )\left({\dot{q}}_{s\tau }(\theta +\tau )-\frac{\partial H}{\partial p_{s\tau }}(\theta +\tau )\right)]d\theta \}dt\\ & \;+\int_{t_{1}-\tau }^{t_{1}}\delta {\dot{q}}_{s}(t)\left\{\lambda (t)p_{s}(t)-\int_{t_{1}-\tau }^{t}\left[-\lambda (\theta )\frac{\partial H}{\partial q_{s}}(\theta )\right]d\theta \right\}dt\\ & \;+\int_{t_{1}-\tau }^{t_{1}}\delta {\dot{\;p}}_{s}(t)\left\{-\int_{t_{1}-\tau }^{t}\left[\lambda (\theta )\left({\dot{q}}_{s}(\theta )-\frac{\partial H}{\partial p_{s}}(\theta )\right)\right]d\theta \right\}dt=0. \end{array}\}$$

According to the basic lemma [15] of the calculus of variation, from equation (3.11), we have

3.12 $$\begin{array}{rl} & \lambda (t)p_{s}(t)+\lambda (t+\tau )p_{s\tau }(t+\tau )+\int_{t}^{t_{1}-\tau }\left[-\lambda (\theta )\frac{\partial H}{\partial q_{s}}(\theta )-\lambda (\theta +\tau )\frac{\partial H}{\partial q_{s\tau }}(\theta +\tau )\right]d\theta =0\\ & \int_{t}^{t_{1}-\tau }[\lambda (\theta )\left({\dot{q}}_{s}(\theta )-\frac{\partial H}{\partial p_{s}}(\theta )\right)+\lambda (\theta +\tau )\left({\dot{q}}_{s\tau }(\theta +\tau )-\frac{\partial H}{\partial p_{s\tau }}(\theta +\tau )\right)]d\theta =0,\;(t_{0}\leq t\leq t_{1}-\tau ;s=1,2,\cdots ,n)\\ & \lambda (t)p_{s}(t)-\int_{t_{1}-\tau }^{t}\left[-\lambda (\theta )\frac{\partial H}{\partial q_{s}}(\theta )\right]d\theta =0,\\ & -\int_{t_{1}-\tau }^{t}\left[\lambda (\theta )\left({\dot{q}}_{s}(\theta )-\frac{\partial H}{\partial p_{s}}(\theta )\right)\right]d\theta =0,\;(t_{1}-\tau <t\leq t_{1};s=1,2,\cdots ,n). \end{array}\}$$

Taking the derivative of equation (3.12) with respect to *t*, we have

3.13 $$\begin{array}{rl} & \lambda (t)\left[{\dot{\;p}}_{s}(t)+\frac{\partial H}{\partial q_{s}}(t)+p_{s}(t)\frac{\partial H}{\partial z}(t)\right]+\lambda (t+\tau )\left[{\dot{\;p}}_{s\tau }(t+\tau )+\frac{\partial H}{\partial q_{s\tau }}(t+\tau )+\;p_{s\tau }(t+\tau )\frac{\partial H}{\partial z}(t+\tau )\right]=0,\\ & \lambda (t)\left[-{\dot{q}}_{s}(t)+\frac{\partial H}{\partial p_{s}}(t)\right]+\lambda (t+\tau )\left[-{\dot{q}}_{s\tau }(t+\tau )+\frac{\partial H}{\partial p_{s\tau }}(t+\tau )\right]=0,\;\;\;(t_{0}\leq t\leq t_{1}-\tau \;;s=1,2,\cdots ,n)\\ & \lambda (t)\left[{\dot{\;p}}_{s}(t)+\frac{\partial H}{\partial q_{s}}(t)+p_{s}(t)\frac{\partial H}{\partial z}(t)\right]=0,\\ & \lambda (t)\left[-{\dot{q}}_{s}(t)+\frac{\partial H}{\partial p_{s}}(t)\right]=0,(t_{1}-\tau <t\leq t_{1};s=1,2,\cdots ,n). \end{array}\}$$

Equation (3.13) can be called the Hamilton canonical equations with time delay based on the variational problem of Herglotz type. A mechanical system whose motion is described by equation (3.13) is called a time-delayed Hamiltonian system of Herglotz type.

## 4. Variation of Hamilton–Herglotz action with time delay

Introduce an *r*-parameter Lie group of transformations with respect to time *t*, generalized coordinates $q_{s}$ and generalized momenta $p_{s}$, i.e.

4.1 $$\bar{t}=t+\Delta t,{\bar{q}}_{s}(\bar{t})=q_{s}(t)+\Delta q_{s},{\bar{\;p}}_{s}(\bar{t})=p_{s}(t)+\Delta p_{s},\;(s=1,2,\cdots ,n)$$

or its expansion

4.2 $$\begin{array}{rl} \bar{t}= & \;t+\varepsilon_{\sigma }\tau^{\sigma }(t,q_{k},p_{k},z),\\ {\bar{q}}_{s}(\bar{t})= & \;q_{s}(t)+\varepsilon_{\sigma }\xi_{s}^{\sigma }(t,q_{k},p_{k},z)\\ \mathrm{and}\;{\bar{\;p}}_{s}(\bar{t})= & \;p_{s}(t)+\varepsilon_{\sigma }\eta_{s}^{\sigma }(t,q_{k},p_{k},z),\;(s,k=1,2,\cdots ,n), \end{array}\}$$

where $\varepsilon_{\sigma }(\sigma =1,2,\cdots ,r)$ are the infinitesimal parameters, $\tau^{\sigma },\xi_{s}^{\sigma }$ and $\eta_{s}^{\sigma }$ are the generators of *r*-parameter Lie group of transformations. Under the action of the transformations (4.2), the Hamilton–Herglotz action *z* with time delay is changed to

4.3 $$\bar{z}(\bar{t})=z(t)+\Delta z(t).$$

For any function *F*, there is a relationship between the non-isochronal variation Δ*F* and the isochronal variation *δF* as follows [16]:

4.4 $$\Delta F=\delta F+\dot{F}\Delta t.$$

As

4.5 $$\frac{d}{dt}\delta F=\delta \dot{F}.$$

Therefore, one obtains

4.6 $$\Delta \dot{F}=\frac{d}{dt}\Delta F-\dot{F}\frac{d}{dt}\Delta t.$$

From equation (2.1), we have

4.7 $$\begin{array}{rl} \Delta \dot{z}= & \;{\dot{q}}_{s}\Delta p_{s}+p_{s}\Delta {\dot{q}}_{s}+{\dot{q}}_{s\tau }\Delta p_{s\tau }+p_{s\tau }\Delta {\dot{q}}_{s\tau }-\frac{\partial H}{\partial t}\Delta t\\ & \;-\frac{\partial H}{\partial q_{s}}\Delta q_{s}-\frac{\partial H}{\partial p_{s}}\Delta p_{s}-\frac{\partial H}{\partial q_{s\tau }}\Delta q_{s\tau }-\frac{\partial H}{\partial p_{s\tau }}\Delta p_{s\tau }-\frac{\partial H}{\partial z}\Delta z. \end{array}\}$$

Using formula (4.6), and considering equation (2.1), we can write equation (4.7) as

4.8 $$\begin{array}{rl} \frac{d}{dt}\Delta z= & {\dot{q}}_{s}\Delta p_{s}+p_{s}\frac{d}{dt}\Delta q_{s}+{\dot{q}}_{s\tau }\Delta p_{s\tau }+p_{s\tau }\frac{d}{dt}\Delta q_{s\tau }-H\frac{d}{dt}\Delta t-\frac{\partial H}{\partial t}\Delta t\\ & -\frac{\partial H}{\partial q_{s}}\Delta q_{s}-\frac{\partial H}{\partial p_{s}}\Delta p_{s}-\frac{\partial H}{\partial q_{s\tau }}\Delta q_{s\tau }-\frac{\partial H}{\partial p_{s\tau }}\Delta p_{s\tau }-\frac{\partial H}{\partial z}\Delta z. \end{array}\}$$

Solving the above equation, we obtain

4.9 $$\begin{array}{rl} \Delta z(t)\lambda (t)-\Delta z(t_{0})= & \int_{t_{0}}^{t}[{\dot{q}}_{s}\Delta p_{s}+p_{s}\frac{d}{dt}\Delta q_{s}+{\dot{q}}_{s\tau }\Delta p_{s\tau }+p_{s\tau }\frac{d}{dt}\Delta q_{s\tau }\\ & -H\frac{d}{dt}\Delta t-\frac{\partial H}{\partial t}\Delta t-\frac{\partial H}{\partial q_{s}}\Delta q_{s}-\frac{\partial H}{\partial p_{s}}\Delta p_{s}-\frac{\partial H}{\partial q_{s\tau }}\Delta q_{s\tau }-\frac{\partial H}{\partial p_{s\tau }}\Delta p_{s\tau }]dt. \end{array}\}$$

It is obvious that $\Delta z(t_{0})=0$. By performing a linear change of variable for the terms involving time delay in the above equation, and using the boundary conditions (2.3), we have

4.10 $$\begin{array}{rl} & \int_{t_{0}}^{t}\lambda (t)[{\dot{q}}_{s\tau }(t)\Delta p_{s\tau }(t)+p_{s\tau }(t)\frac{d}{dt}\Delta q_{s\tau }(t)-\frac{\partial H}{\partial q_{s\tau }}(t)\Delta q_{s\tau }(t)-\frac{\partial H}{\partial p_{s\tau }}(t)\Delta p_{s\tau }(t)]dt\\ & \;=\int_{t_{0}-\tau }^{t-\tau }\lambda (\varphi +\tau )[{\dot{q}}_{s\tau }(\varphi +\tau )\Delta p_{s\tau }(\varphi +\tau )+p_{s\tau }(\varphi +\tau )\frac{d}{dt}\Delta q_{s\tau }(\varphi +\tau )\\ & \;-\frac{\partial H}{\partial q_{s\tau }}(\varphi +\tau )\Delta q_{s\tau }(\varphi +\tau )-\frac{\partial H}{\partial p_{s\tau }}(\varphi +\tau )\Delta p_{s\tau }(\varphi +\tau )]d\varphi \\ & \;=\int_{t_{0}}^{t-\tau }\lambda (\varphi +\tau )[{\dot{q}}_{s\tau }(\varphi +\tau )\Delta p_{s}(\varphi )+p_{s\tau }(\varphi +\tau )\frac{d}{dt}\Delta q_{s}(\varphi )\\ & \;-\frac{\partial H}{\partial q_{s\tau }}(\varphi +\tau )\Delta q_{s}(\varphi )-\frac{\partial H}{\partial p_{s\tau }}(\varphi +\tau )\Delta p_{s}(\varphi )]d\varphi . \end{array}\}$$

Substituting equation (4.10) into equation (4.9), we obtain

4.11 $$\begin{array}{rl} \Delta z(t)\;\lambda (t)= & \int_{t_{0}}^{t-\tau }\{\lambda (t)\left[{\dot{q}}_{s}(t)\Delta p_{s}+p_{s}(t)\frac{d}{dt}\Delta q_{s}-H(t)\frac{d}{dt}\Delta t-\frac{\partial H}{\partial t}(t)\Delta t-\frac{\partial H}{\partial q_{s}}(t)\Delta q_{s}-\frac{\partial H}{\partial p_{s}}(t)\Delta p_{s}\right]\\ & +\lambda (t+\tau )[{\dot{q}}_{s\tau }(t+\tau )\Delta p_{s}+p_{s\tau }(t+\tau )\frac{d}{dt}\Delta q_{s}-\frac{\partial H}{\partial q_{s\tau }}(t+\tau )\Delta q_{s}-\frac{\partial H}{\partial p_{s\tau }}(t+\tau )\Delta p_{s}]\}dt\\ & +\int_{t-\tau }^{t}\lambda (t)[{\dot{q}}_{s}(t)\Delta p_{s}+p_{s}(t)\frac{d}{dt}\Delta q_{s}-H(t)\frac{d}{dt}\Delta t-\frac{\partial H}{\partial t}(t)\Delta t-\frac{\partial H}{\partial q_{s}}(t)\Delta q_{s}-\frac{\partial H}{\partial p_{s}}(t)\Delta p_{s}]dt. \end{array}\}$$

Equation (4.9) can also be expressed as

4.12 $$\begin{array}{rl} \Delta z(t)\lambda (t)= & \int_{t_{0}}^{t}\{\frac{d}{dt}[\lambda (t)\left(\;p_{s}\Delta q_{s}+p_{s\tau }\Delta q_{s\tau }-H\Delta t\right)]\\ & \;+\lambda (t)[\left(-{\dot{\;p}}_{s}-\frac{\partial H}{\partial q_{s}}-p_{s}\frac{\partial H}{\partial z}\right)(\Delta q_{s}-{\dot{q}}_{s}\Delta t)+\left({\dot{q}}_{s}-\frac{\partial H}{\partial p_{s}}\right)(\Delta p_{s}-{\dot{\;p}}_{s}\Delta t)]\\ & \;+\lambda (t)[\left(-{\dot{\;p}}_{s\tau }-\frac{\partial H}{\partial q_{s\tau }}-p_{s\tau }\frac{\partial H}{\partial z}\right)(\Delta q_{s\tau }-{\dot{q}}_{s\tau }\Delta t)+\left({\dot{q}}_{s\tau }-\frac{\partial H}{\partial p_{s\tau }}\right)(\Delta p_{s\tau }-{\dot{\;p}}_{s\tau }\Delta t)]\}dt. \end{array}\}$$

By performing a linear change of variable for the terms involving time delay in equation (4.12), and using the boundary conditions (2.3), we have

4.13 $$\begin{array}{rl} \int_{t_{0}}^{t}\frac{d}{dt}[\lambda (t)p_{s\tau }(t)\Delta q_{s\tau }(t)]dt= & \int_{t_{0}-\tau }^{t-\tau }\frac{d}{d\varphi }[\lambda (\varphi +\tau )p_{s\tau }(\varphi +\tau )\Delta q_{s\tau }(\varphi +\tau )]d\varphi \\ = & \int_{t_{0}}^{t-\tau }\frac{d}{d\varphi }[\lambda (\varphi +\tau )p_{s\tau }(\varphi +\tau )\Delta q_{s}(\varphi )]d\varphi \end{array}\}$$

and

4.14 $$\begin{array}{rl} & \int_{t_{0}}^{t}\lambda (t)[\left(-{\dot{\;p}}_{s\tau }(t)-\frac{\partial H}{\partial q_{s\tau }}(t)-p_{s\tau }(t)\frac{\partial H}{\partial z}(t)\right)(\Delta q_{s\tau }(t)-{\dot{q}}_{s\tau }(t)\Delta t)\\ & \;+\left({\dot{q}}_{s\tau }(t)-\frac{\partial H}{\partial p_{s\tau }}(t)\right)(\Delta p_{s\tau }(t)-{\dot{\;p}}_{s\tau }(t)\Delta t)]dt\\ & \;=\int_{t_{0}-\tau }^{t-\tau }\lambda (\varphi +\tau )[(-{\dot{\;p}}_{s\tau }(\varphi +\tau )-\frac{\partial H}{\partial q_{s\tau }}(\varphi +\tau )-p_{s\tau }(\varphi +\tau )\frac{\partial H}{\partial z}(\varphi +\tau ))(\Delta q_{s\tau }(\varphi +\tau )\\ & \;-{\dot{q}}_{s\tau }(\varphi +\tau )\Delta \varphi )+\left({\dot{q}}_{s\tau }(\varphi +\tau )-\frac{\partial H}{\partial p_{s\tau }}(\varphi +\tau )\right)\left(\Delta p_{s\tau }(\varphi +\tau )-{\dot{\;p}}_{s\tau }(\varphi +\tau )\Delta \varphi \right)]d\varphi \\ & \;=\int_{t_{0}}^{t-\tau }\lambda (\varphi +\tau )[(-{\dot{\;p}}_{s\tau }(\varphi +\tau )-\frac{\partial H}{\partial q_{s\tau }}(\varphi +\tau )-p_{s\tau }(\varphi +\tau )\frac{\partial H}{\partial z}(\varphi +\tau ))(\Delta q_{s}(\varphi )\\ & \;-{\dot{q}}_{s}(\varphi )\Delta \varphi )+\left({\dot{q}}_{s\tau }(\varphi +\tau )-\frac{\partial H}{\partial p_{s\tau }}(\varphi +\tau )\right)\left(\Delta p_{s}(\varphi )-{\dot{\;p}}_{s}(\varphi )\Delta \varphi \right)]d\varphi . \end{array}\}$$

Substituting formulae (4.13) and (4.14) into equation (4.12), we have

4.15 $$\begin{array}{rl} & \Delta z(t)\lambda (t)=\int_{t_{0}}^{t-\tau }\{\frac{d}{dt}[\lambda (t)(p_{s}(t)\Delta q_{s}-H(t)\Delta t(+\lambda (t+\tau )p_{s\tau }(t+\tau )\Delta q_{s}]\\ & \;+\lambda (t)[\left(-{\dot{\;p}}_{s}(t)-\frac{\partial H}{\partial q_{s}}(t)-p_{s}(t)\frac{\partial H}{\partial z}(t)\right)(\Delta q_{s}-{\dot{q}}_{s}(t)\Delta t)+\left(\;{\dot{q}}_{s}(t)-\frac{\partial H}{\partial p_{s}}(t)\right)(\Delta p_{s}-\;{\dot{p}}_{s}(t)\Delta t)]\\ & \;+\lambda (t+\tau )[\left(-{\dot{\;p}}_{s\tau }(t+\tau )-\frac{\partial H}{\partial q_{s\tau }}(t+\tau )-p_{s\tau }(t+\tau )\frac{\partial H}{\partial z}(t+\tau )\right)(\Delta q_{s}-{\dot{q}}_{s}(t)\Delta t)\\ & \;+\left(q_{s\tau }(t+\tau )-\frac{\partial H}{\partial p_{s\tau }}(t+\tau )\right)\left(\Delta p_{s}-{\dot{\;p}}_{s}(t)\Delta t\right)]\}dt+\int_{t-\tau }^{t}\{\frac{d}{dt}[\lambda (t)(\;p_{s}(t)\Delta q_{s}-H(t)\Delta t)]\\ & \;+\lambda (t)[(-{\dot{\;p}}_{s}(t)-\frac{\partial H}{\partial q_{s}}(t)-p_{s}(t)\frac{\partial H}{\partial z}(t))(\Delta q_{s}-{\dot{q}}_{s}(t)\Delta t)\\ & \;+\left({\dot{q}}_{s}(t)-\frac{\partial H}{\partial p_{s}}(t)\right)(\Delta p_{s}-{\dot{\;p}}_{s}(t)\Delta t)]\}dt. \end{array}\}$$

Owing to

4.16 $$\Delta t=\varepsilon_{\sigma }\tau^{\sigma },\;\Delta q_{s}=\varepsilon_{\sigma }\xi_{s}^{\sigma },\;\Delta p_{s}=\varepsilon_{\sigma }\eta_{s}^{\sigma }\;(s=1,2,\cdots ,n).$$

Substituting formulae (4.16) into equations (4.11) and (4.15), we obtain

4.17 $$\begin{array}{rl} \Delta z(t)\lambda (t)= & \int_{t_{0}}^{t-\tau }\{\lambda (t)\left[{\dot{q}}_{s}(t)\eta_{s}^{\sigma }+p_{s}(t){\dot{\xi }}_{s}^{\sigma }-H(t){\dot{\tau }}^{\sigma }-\frac{\partial H}{\partial t}(t)\tau^{\sigma }-\frac{\partial H}{\partial q_{s}}(t)\xi_{s}^{\sigma }-\frac{\partial H}{\partial p_{s}}(t)\eta_{s}^{\sigma }\right]\\ & \;+\lambda (t+\tau )\left[{\dot{q}}_{s\tau }(t+\tau )\eta_{s}^{\sigma }+p_{s\tau }(t+\tau ){\dot{\xi }}_{s}^{\sigma }-\frac{\partial H}{\partial q_{s\tau }}(t+\tau )\xi_{s}^{\sigma }-\frac{\partial H}{\partial p_{s\tau }}(t+\tau )\eta_{s}^{\sigma }\right]\}\varepsilon_{\sigma }dt\\ & \;+\int_{t-\tau }^{t}\lambda (t)\left[{\dot{q}}_{s}(t)\eta_{s}^{\sigma }+p_{s}(t){\dot{\xi }}_{s}^{\sigma }-H(t){\dot{\tau }}^{\sigma }-\frac{\partial H}{\partial t}(t)\tau^{\sigma }-\frac{\partial H}{\partial q_{s}}(t)\xi_{s}^{\sigma }-\frac{\partial H}{\partial p_{s}}(t)\eta_{s}^{\sigma }\right]\varepsilon_{\sigma }dt \end{array}\}$$

and

4.18 $$\begin{array}{rl} & \Delta z(t)\lambda (t)=\int_{t_{0}}^{t-\tau }\{\frac{d}{dt}[\lambda (t)(\;p_{s}(t)\xi_{s}^{\sigma }-H(t)\tau^{\sigma })+\lambda (t+\tau )p_{s\tau }(t+\tau )\xi_{s}^{\sigma }\;]\\ & \;+\lambda (t)\left[\left(-{\dot{\;p}}_{s}(t)-\frac{\partial H}{\partial q_{s}}(t)-p_{s}(t)\frac{\partial H}{\partial z}(t)\right)(\xi_{s}^{\sigma }-{\dot{q}}_{s}(t)\tau^{\sigma })+\left({\dot{q}}_{s}(t)-\frac{\partial H}{\partial p_{s}}(t)\right)(\eta_{s}^{\sigma }-{\dot{\;p}}_{s}(t)\tau^{\sigma })\right]\\ & \;+\lambda (t+\tau )[\left(-{\dot{\;p}}_{s\tau }(t+\tau )-\frac{\partial H}{\partial q_{s\tau }}(t+\tau )-p_{s\tau }(t+\tau )\frac{\partial H}{\partial z}(t+\tau )\right)(\xi_{s}^{\sigma }-{\dot{q}}_{s}(t)\tau^{\sigma })\\ & \;+\left({\dot{q}}_{s\tau }(t+\tau )-\frac{\partial H}{\partial p_{s\tau }}(t+\tau )\right)(\eta_{s}^{\sigma }-\overset{.}{\underset{s}{\;p}}(t)\tau^{\sigma })]\}\varepsilon_{\sigma }dt\\ & \;+\int_{t-\tau }^{t}\{\frac{d}{dt}[\lambda (t)(\;p_{s}(t)\xi_{s}^{\sigma }-H(t)\tau^{\sigma })]+\lambda (t)[\left(-{\dot{\;p}}_{s}(t)-\frac{\partial H}{\partial q_{s}}(t)-p_{s}(t)\frac{\partial H}{\partial z}(t)\right)\\ & \;\times (\xi_{s}^{\sigma }-{\dot{q}}_{s}(t)\tau^{\sigma })+\left({\dot{q}}_{s}(t)-\frac{\partial H}{\partial p_{s}}(t)\right)(\eta_{s}^{\sigma }-{\dot{\;p}}_{s}(t)\tau^{\sigma })]\}\varepsilon_{\sigma }dt. \end{array}\}$$

Formulae (4.17) and (4.18) are the basic formulae of variation for the Hamilton–Herglotz action with time delay.

## 5. Definition and criterion of Noether's symmetry of a time-delayed Hamiltonian system of Herglotz type

The classical Noether symmetry refers to the invariance of Hamilton action under the infinitesimal transformation with respect to the generalized coordinates and time. In this section, we establish the definition and the criterion of the Noether symmetry of a time-delayed Hamiltonian system of Herglotz type.

**Definition 5.1.**

For the variational problem of Herglotz type with time delay, if its Hamilton–Herglotz action is an invariant under the infinitesimal transformations (4.2) of *r*-parameter Lie group of transformations, that is, for each of the infinitesimal transformations (4.2), the formula

5.1 $$\Delta z(t_{1})=0,$$

always holds, then the infinitesimal transformations (4.2) are called the Noether symmetry transformations of the time-delayed Hamiltonian system (3.13) of Herglotz type.

By definition (5.1) and formula (4.17), the following criterion can be obtained:

**Criterion 5.2.**

For the infinitesimal transformations (4.2) of *r*-parameter Lie group of transformations, if the generators $\tau^{\sigma },\xi_{s}^{\sigma },\eta_{s}^{\sigma }$ satisfy the following conditions:

5.2 $$\begin{array}{rl} & \lambda (t)\left[{\dot{q}}_{s}(t)\eta_{s}^{\sigma }+p_{s}(t){\dot{\xi }}_{s}^{\sigma }-H(t){\dot{\tau }}^{\sigma }-\frac{\partial H}{\partial t}(t)\tau^{\sigma }-\frac{\partial H}{\partial q_{s}}(t)\xi_{s}^{\sigma }-\frac{\partial H}{\partial p_{s}}(t)\eta_{s}^{\sigma }\right]\\ & +\lambda (t+\tau )\left[{\dot{q}}_{s\tau }(t+\tau )\eta_{s}^{\sigma }+p_{s\tau }(t+\tau ){\dot{\xi }}_{s}^{\sigma }-\frac{\partial H}{\partial q_{s\tau }}(t+\tau )\xi_{s}^{\sigma }-\frac{\partial H}{\partial p_{s\tau }}(t+\tau )\eta_{s}^{\sigma }\right]=0,\;(\sigma =1,2,\cdots ,n). \end{array}\}$$

for $t_{0}\leq t\leq t_{1}-\tau$, and

5.3 $$\lambda (t)\left[{\dot{q}}_{s}(t)\eta_{s}^{\sigma }+p_{s}(t){\dot{\xi }}_{s}^{\sigma }-H(t){\dot{\tau }}^{\sigma }-\frac{\partial H}{\partial t}(t)\tau^{\sigma }-\frac{\partial H}{\partial q_{s}}(t)\xi_{s}^{\sigma }-\frac{\partial H}{\partial p_{s}}(t)\eta_{s}^{\sigma }\right]=0,\;(\sigma =1,2,\cdots ,n),$$

for $t_{1}-\tau \leq t\leq t_{1}$, then the transformations (4.2) are the Noether symmetry transformations of the time-delayed Hamiltonian system (3.13) of Herglotz type.

## 6. Noether's theorem of a time-delayed Hamiltonian system of Herglotz type

Noether's theorem reveals the inherent relation between the Noether symmetry and the conserved quantity [16,17]. Recently, a series of important advances have been made in the research of Noether symmetry and conserved quantity [18–36]. In this section, we establish Noether's theorem for the time-delayed Hamiltonian system of Herglotz type. There are

**Theorem 6.1.**

*For the time-delayed Hamiltonian system (3.13) of Herglotz type, if the infinitesimal transformations (4.2) of* *r-parameter Lie group of transformations are the Noether symmetry transformations, then the system exists with* *r* *independent conserved quantities, which are*

6.1 $$I^{\sigma }=\lambda (t)(\;p_{s}(t)\xi_{s}^{\sigma }-H(t)\tau^{\sigma })+\lambda (t+\tau )p_{s\tau }(t+\tau )\xi_{s}^{\sigma }=c^{\sigma },\;(\sigma =1,2,\cdots ,r),$$

*for* $t_{0}\leq t\leq t_{1}-\tau$, *and*

6.2 $$I^{\sigma }=\lambda (t)(\;p_{s}(t)\xi_{s}^{\sigma }-H(t)\tau^{\sigma })=c^{\sigma },\;(\sigma =1,2,\cdots ,r).$$

*for* $t_{1}-\tau <t\leq t_{1}$, *here* $\lambda (t)=\exp\left(\int_{t_{0}}^{t}(\partial H/\partial z)d\theta \right)$.

*Proof.* Because the infinitesimal transformations (4.2) of *r*-parameter Lie group of transformations are the Noether symmetry transformations of the system (3.13), by definition (5.1), we have

$$\Delta z(t_{1})=0.$$

Substituting the above formula into formula (4.18), we obtain

6.3 $$\begin{array}{rl} & \int_{t_{0}}^{t_{1}-\tau }\{\frac{d}{dt}[\lambda (t)(\;p_{s}(t)\xi_{s}^{\sigma }-H(t)\tau^{\sigma })+\lambda (t+\tau )p_{s\tau }(t+\tau )\xi_{s}^{\sigma }]\\ & +\lambda (t)\left[\left(-{\dot{\;p}}_{s}(t)-\frac{\partial H}{\partial q_{s}}(t)-p_{s}(t)\frac{\partial H}{\partial z}(t)\right)(\xi_{s}^{\sigma }-{\dot{q}}_{s}(t)\tau^{\sigma })+\left({\dot{q}}_{s}(t)-\frac{\partial H}{\partial p_{s}}(t)\right)(\eta_{s}^{\sigma }-{\dot{\;p}}_{s}(t)\tau^{\sigma })\right]\\ & +\lambda (t+\tau )[\left(-{\dot{\;p}}_{s\tau }(t+\tau )-\frac{\partial H}{\partial q_{s\tau }}(t+\tau )-p_{s\tau }(t+\tau )\frac{\partial H}{\partial z}(t+\tau )\right)(\xi_{s}^{\sigma }-{\dot{q}}_{s}(t)\tau^{\sigma })\\ & +\left({\dot{q}}_{s\tau }(t+\tau )-\frac{\partial H}{\partial p_{s\tau }}(t+\tau )\right)(\eta_{s}^{\sigma }-{\dot{\;p}}_{s}(t)\tau^{\sigma })]\varepsilon_{\sigma }dt\\ & +\int_{t_{1}-\tau }^{t_{1}}\{\frac{d}{dt}[\lambda (t)(\;p_{s}(t)\xi_{s}^{\sigma }-H(t)\tau^{\sigma })]+\lambda (t)[\left(-{\dot{\;p}}_{s}(t)-\frac{\partial H}{\partial q_{s}}(t)-p_{s}(t)\frac{\partial H}{\partial z}(t)\right)\\ & \cdot (\xi_{s}^{\sigma }-{\dot{q}}_{s}(t)\tau^{\sigma })+\left({\dot{q}}_{s}(t)-\frac{\partial H}{\partial p_{s}}(t)\right)(\eta_{s}^{\sigma }-{\dot{\;p}}_{s}(t)\tau^{\sigma })]\}\varepsilon_{\sigma }dt=0. \end{array}\}$$

Substituting equations (3.13) into formula (6.3), we have

6.4 $$\begin{array}{rl} & \int_{t_{0}}^{t_{1}-\tau }\frac{d}{dt}[\lambda (t)(\;p_{s}(t)\xi_{s}^{\sigma }-H(t)\tau^{\sigma })+\lambda (t+\tau )p_{s\tau }(t+\tau )\xi_{s}^{\sigma }]\varepsilon_{\sigma }dt\\ & +\int_{t_{1}-\tau }^{t_{1}}\frac{d}{dt}[\lambda (t)(\;p_{s}(t)\xi_{s}^{\sigma }-H(t)\tau^{\sigma })]\varepsilon_{\sigma }dt=0. \end{array}\}$$

From the independence of $\varepsilon_{\sigma }$ and the arbitrariness of the integral interval $\left[t_{0},t_{1}\right]$, we obtain

6.5 $$\frac{d}{dt}[\lambda (t)(\;p_{s}(t)\xi_{s}^{\sigma }-H(t)\tau^{\sigma })+\lambda (t+\tau )p_{s\tau }(t+\tau )\xi_{s}^{\sigma }]=0,\;(t_{0}\leq t\leq t_{1}-\tau )$$

and

6.6 $$\frac{d}{dt}[\lambda (t)(\;p_{s}(t)\xi_{s}^{\sigma }-H(t)\tau^{\sigma })]=0,\;(t_{1}-\tau <t\leq t_{1}),$$

where σ=1,2,⋯,r. By integrating equations (6.5) and (6.6), we can obtain the conserved quantities (6.1) and (6.2) immediately. Therefore, the theorem is proved.

Theorem (6.1) can be called Noether's theorem for the time-delayed Hamiltonian system of Herglotz type. By using the theorem, we can obtain a conserved quantity of a time-delayed Hamiltonian system of Herglotz type if a Noether symmetry of the system is found. As a non-conservative or dissipative system can be reduced to a variational problem of Herglotz type, and therefore, the conserved quantity of a non-conservative or dissipative system can be found by using theorem (6.1).

## 7. Some special cases

In this section, we discuss two special cases.

**Case 7.1.**

The time-delayed Hamiltonian system based on the classical variational problem.

If the Hamiltonian *H* does not contain z(t), then equation (2.1) is reduced to

7.1 $$z=\int_{t_{0}}^{t_{1}}[\;p_{s}(t){\dot{q}}_{s}(t)+p_{s}(t-\tau ){\dot{q}}_{s}(t-\tau )-H(t,q_{s}(t),p_{s}(t),q_{s}(t-\tau ),p_{s}(t-\tau ))]dt.$$

So, equation (3.13) becomes

7.2 $$\begin{array}{rl} & {\dot{\;p}}_{s}(t)+\frac{\partial H}{\partial q_{s}}(t)+{\dot{\;p}}_{s\tau }(t+\tau )+\frac{\partial H}{\partial q_{s\tau }}(t+\tau )=0,\\ & -{\dot{q}}_{s}(t)+\frac{\partial H}{\partial p_{s}}(t)-{\dot{q}}_{s\tau }(t+\tau )+\frac{\partial H}{\partial p_{s\tau }}(t+\tau )=0,\;(t_{0}\leq t\leq t_{1}-\tau ;s=1,2,\cdots ,n)\\ \;\mathrm{and}\; & {\dot{p}}_{s}(t)+\frac{\partial H}{\partial q_{s}}(t)=0,-{\dot{q}}_{s}(t)+\frac{\partial H}{\partial p_{s}}(t)=0,\;(t_{1}-\tau <t\leq t_{1};s=1,2,\cdots ,n). \end{array}\}$$

These are the differential equations of motion for the time-delayed Hamiltonian system based on the classical variational problem [36].

The *r*-parameter Lie group of transformations (4.2) become

7.3 $$\begin{array}{rl} \bar{t}=\; & t+\varepsilon_{\sigma }\tau^{\sigma }(t,q_{k},p_{k}),\\ {\bar{q}}_{s}(\bar{t})=\; & q_{s}(t)+\varepsilon_{\sigma }\xi_{s}^{\sigma }(t,q_{k},p_{k})\\ \;\mathrm{and}\;{\bar{\;p}}_{s}(\bar{t})=\; & p_{s}(t)+\varepsilon_{\sigma }\eta_{s}^{\sigma }(t,q_{k},p_{k}),\;(s,k=1,2,\cdots ,n). \end{array}\}$$

And the criterion (5.2) and theorem (6.1) become

**Criterion 7.2.**

For the infinitesimal transformations (7.3) of *r*-parameter Lie group of transformations, if the generators $\tau^{\sigma },\xi_{s}^{\sigma },\eta_{s}^{\sigma }$ satisfy the following conditions

7.4 $$\begin{array}{rl} & [{\dot{q}}_{s}(t)+{\dot{q}}_{s\tau }(t+\tau )]\eta_{s}^{\sigma }+[\;p_{s}(t)+p_{s\tau }(t+\tau )]{\dot{\xi }}_{s}^{\sigma }-H(t){\dot{\tau }}^{\sigma }-\frac{\partial H}{\partial t}(t)\tau^{\sigma }\\ & -\left[\frac{\partial H}{\partial q_{s}}(t)+\frac{\partial H}{\partial q_{s\tau }}(t+\tau )\right]\xi_{s}^{\sigma }-\left[\frac{\partial H}{\partial p_{s}}(t)+\frac{\partial H}{\partial p_{s\tau }}(t+\tau )\right]\eta_{s}^{\sigma }=0,\;(\sigma =1,2,\cdots ,r). \end{array}\}$$

for $t_{0}\leq t\leq t_{1}-\tau$, and

7.5 $${\dot{q}}_{s}(t)\eta_{s}^{\sigma }+p_{s}(t){\dot{\xi }}_{s}^{\sigma }-H(t){\dot{\tau }}^{\sigma }-\frac{\partial H}{\partial t}(t)\tau^{\sigma }-\frac{\partial H}{\partial q_{s}}(t)\xi_{s}^{\sigma }-\frac{\partial H}{\partial p_{s}}(t)\eta_{s}^{\sigma }=0,\;(\sigma =1,2,\cdots ,r).$$

for $t_{1}-\tau <t\leq t_{1}$, then the transformations (7.3) are the Noether symmetry transformations of the time-delayed Hamiltonian system (7.2) based on the classical variational problem.

**Theorem 7.3.**

*For the time-delayed Hamiltonian system (7.2) based on the classical variational problem, if the infinitesimal transformations (7.3) of* *r-parameter Lie group of transformations are the Noether symmetry transformations, then the system exists with* *r* *independent conserved quantities, which are*

7.6 $$I^{\sigma }=[\;p_{s}(t)+p_{s\tau }(t+\tau )]\xi_{s}^{\sigma }-H(t)\tau^{\sigma }=c^{\sigma },\;(\sigma =1,2,\cdots ,r).$$

*for* $t_{0}\leq t\leq t_{1}-\tau$, *and*

7.7 $$I^{\sigma }=p_{s}(t)\xi_{s}^{\sigma }-H(t)\tau^{\sigma }=c^{\sigma },\;(\sigma =1,2,\cdots ,r),$$

*for* $t_{1}-\tau <t\leq t_{1}$.

Theorem (7.3) is Noether's theorem of a time-delayed Hamiltonian system based on the classical variational problem, which was presented in [36].

**Case 7.4.**

The Hamiltonian system based on the variational problem of Herglotz type.

If no time delay exists in the system, then equation (2.1) is reduced to

7.8 $$\dot{z}(t)=p_{s}(t){\dot{q}}_{s}(t)-H(t,q_{s}(t),p_{s}(t),z(t)).$$

Equation (3.13) becomes

7.9 $$\begin{array}{rl} & \lambda (t)\left[{\dot{\;p}}_{s}(t)+\frac{\partial H}{\partial q_{s}}(t)+p_{s}(t)\frac{\partial H}{\partial z}(t)\right]=0,\\ & \lambda (t)\left[-{\dot{q}}_{s}(t)+\frac{\partial H}{\partial p_{s}}(t)\right]=0,\;(s=1,2,\cdots ,n). \end{array}\}$$

Equation (7.9) is the Hamilton canonical equation for the variational problem of Herglotz type.

Criterion (5.2) and theorem (6.1) become

**Criterion 7.5.**

For the infinitesimal transformations (4.2) of *r*-parameter Lie group of transformations, if the generators $\tau^{\sigma },\xi_{s}^{\sigma },\eta_{s}^{\sigma }$ satisfy the following conditions

7.10 $${\dot{q}}_{s}(t)\eta_{s}^{\sigma }+p_{s}(t){\dot{\xi }}_{s}^{\sigma }-H(t){\dot{\tau }}^{\sigma }-\frac{\partial H}{\partial t}(t)\tau^{\sigma }-\frac{\partial H}{\partial q_{s}}(t)\xi_{s}^{\sigma }-\frac{\partial H}{\partial p_{s}}(t)\eta_{s}^{\sigma }=0,(\sigma =1,2,\cdots ,r)$$

then the transformations (4.2) are the Noether symmetry transformations for the variational problem of Herglotz type.

**Theorem 7.6.**

*For the Hamiltonian system based on the variational problem of Herglotz type, if the infinitesimal transformations (4.2) of* *r-parameter Lie group of transformations are the Noether symmetry transformations of the system (7.9), then the system exists with* *r* *independent conserved quantities, which are*

7.11 $$I^{\sigma }=\lambda (t)(\;p_{s}(t)\xi_{s}^{\sigma }-H(t)\tau^{\sigma })=c^{\sigma },\;(\sigma =1,2,\cdots ,r),$$

*where* $\lambda (t)=\exp\left(\int_{t_{0}}^{t}(\partial H/\partial z)d\theta \right)$.

Theorem (7.6) is Noether's theorem for the Hamiltonian system based on the variational problem of Herglotz type.

## 8. Example

For example, let us consider a time-delayed Hamiltonian system of Herglotz type. Its Hamiltonian is

8.1 $$H=\frac{1}{2}[{\left(q(t)\right)}^{2}+{\left(\;p(t)\right)}^{2}+{\left(\;p(t-\tau )\right)}^{2}]+z(t),$$

where the Hamilton–Herglotz action z(t) satisfies

8.2 $$\dot{z}(t)=p(t)\dot{q}(t)+p(t-\tau )\dot{q}(t-\tau )-\frac{1}{2}[{\left(q(t)\right)}^{2}+{\left(\;p(t)\right)}^{2}+{\left(\;p(t-\tau )\right)}^{2}]-z(t).$$

Equation (3.13) gives

8.3 $$\begin{array}{rl} & \lambda (t)[\dot{\;p}(t)+q(t)+p(t)]+\lambda (t+\tau )[{\dot{\;p}}_{\tau }(t+\tau )+p_{\tau }(t+\tau )]=0,\\ & \lambda (t)[-\dot{q}(t)+p(t)]+\lambda (t+\tau )[-{\dot{q}}_{\tau }(t+\tau )+q_{\tau }(t+\tau )]=0,\;(t_{0}\leq t\leq t_{1}-\tau ),\\ & \lambda (t)[\dot{\;p}(t)+q(t)+p(t)]=0,\lambda (t)[-\dot{q}(t)+p(t)]=0,\;(t_{1}-\tau <t\leq t_{1}), \end{array}\}$$

where $\lambda =e^{t-t_{0}}$. According to criterion (5.2), when $t_{0}\leq t\leq t_{1}-\tau$, the criterion equation (5.2) gives

8.4 $$\begin{array}{rl} & \lambda (t)[\dot{q}(t)\eta +p(t)\dot{\xi }-H\dot{\tau }-q(t)\xi -p(t)\eta ]\\ & +\lambda (t+\tau )[{\dot{q}}_{\tau }(t+\tau )\eta +p_{\tau }(t+\tau )\dot{\xi }-p_{\tau }(t+\tau )\eta ]=0. \end{array}\}$$

Equation (8.4) has a solution

8.5 $$\tau =0,\xi =q(t)+p(t)+\frac{q^{2}(t)}{\left(1+e^{\tau })p(t\right)},\eta =1.$$

When $t_{1}-\tau <t\leq t_{1}$, the criterion equation (5.3) gives

8.6 $$\lambda (t)[\dot{q}(t)\eta +p(t)\dot{\xi }-H\dot{\tau }-q(t)\xi -p(t)\eta ]=0.$$

Equation (8.6) has a solution

8.7 $$\tau =0,\xi =q(t)+p(t)+\frac{q^{2}(t)}{\;p(t)},\eta =t.$$

The generators (8.5) and (8.7) correspond to the Noether symmetry of the time-delayed Hamiltonian system of Herglotz type under study. By Theorem (6.1), we obtain

8.8 $$I=e^{t-t_{0}}[q^{2}(t)+(1+e^{\tau })(q(t)p(t)+p^{2}(t))]=\mathrm{const}.$$

for $t_{0}\leq t\leq t_{1}-\tau$, and

8.9 $$I=e^{t-t_{0}}[q^{2}(t)+q(t)p(t)+p^{2}(t)]=\mathrm{const}.$$

for $t_{1}-\tau <t\leq t_{1}$. The formulae (8.8) and (8.9) are the conserved quantities led by the Noether symmetries of the system.

## 9. Conclusion

The generalized variational principle of Herglotz provides an effective way to study the dynamics of non-conservative or dissipative systems. In this paper, we studied the variational problem of Herglotz type with time delay and proved Noether's theorem for a time-delayed Hamiltonian system. The main contributions of this paper lie in: the first is that it puts forward the variational problem of Herglotz type with time delay, and gives a variational description of the time-delayed Hamiltonian system and establishes the Hamilton canonical equations of the system. The second is that it derives two basic formulae of variation for the Hamilton–Herglotz action with time delay. The third is that it establishes the definition and criterion of the Noether symmetry for the time-delayed Hamiltonian system of Herglotz type, and proves Noether's theorem of the system. Noether's theorem for a time-delayed Hamiltonian system based on the classical variational problem and Noether's theorem for a Hamiltonian system based on the variational problem of Herglotz type are special cases of our results. The methods and results of this paper can be further extended to the nonholonomic constrained mechanical system etc.

## Data accessibility

This article has no additional data.
